# Post-translational regulation of a *Porphyromonas gingivalis* regulator

**DOI:** 10.1080/20002297.2018.1487743

**Published:** 2018-07-03

**Authors:** Yuqing Li, Karthik Krishnan, Margaret J. Duncan

**Affiliations:** a Department of Microbiology, The Forsyth Institute, Cambridge, MA, USA; b State Key Laboratory of Oral Diseases, National Clinical Research Center for Oral Diseases, West China Hospital of Stomatology, Sichuan University, Chengdu, PR China; c Office of Dean of Research and Graduate Studies, Shiv Nadar University, Gautam Buddha Nagar, India

**Keywords:** *Porphyromonas gingivalis*, RprY, acetylation, CobB, oxidative response

## Abstract

**Background**: Bacteria use two-component signal transduction systems (among others) to perceive and respond to environmental changes. Within the genus *Porphyromonas*, we observed degeneration of these systems, as exemplified by the loss of RprX, the sensor kinase partner of the RprY.

**Objective**: The purpose of this study was to investigate modulation of RprY function by acetylation.

**Design**: The transcriptional activity of the *rprY-pat* genes were measured by RT-PCR and 5ʹ-RACE. The acetylation of RprY were detected by western blotting. Electromobility shift and *in vitro* ChIP assays were used to measure the DNA binding activity of RprY. The expression of RprY target genes was measured by qRT-PCR. Effects of acetylation on phosphorylation of RprY were measured by Phos-tag gels.

**Results**: The *rprY* gene is cotranscribed with *pat*. RprY is acetylated *in vivo*, and autoacetylated *in vitro* in a reaction that is enhanced by Pat; the CobB sirtuin deacetylates RprY. Acetylation reduced the DNA binding of RprY. Induced oxidative stress decreased production of RprY *in vivo*, increased its acetylation and increased expression of *nqrA*.

**Conclusions**: We propose that to compensate for the loss of RprX, *P. gingivalis* has evolved a novel mechanism to inactivate RprY through acetylation.

## Introduction

*Porphyromonas gingivalis*, a Gram-negative oral anaerobe, is associated with adult periodontitis which affects almost 50% of the US population [1]. In the gingival crevice, anaerobes such as *P. gingivalis* respond to environmental signals, some yet unknown, which enhance growth of the organism and induce virulence factors that lead to gingivitis and periodontal disease. The ability of bacteria to respond to environmental changes is in part regulated by two-component signal transduction systems [2]. The simplest system involves a sensor histidine kinase (HK), most often a transmembrane protein with its N-terminus located in the periplasm to monitor environmental changes. Upon receiving the appropriate cue, the HK autophosphorylates a conserved histidine residue in the cytoplasmic C-terminus. In a phospho-relay, the HK functions as phosphoryl donor to a conserved aspartate residue in the cognate response regulator protein (RR), inducing change to an active conformation that, when acting as a transcription factor, can bind to target gene promoter sequences. Response regulators can activate and/or repress transcription of their target genes.

In a previous report, we presented data indicating that the response regulator RprY of *P. gingivalis* strain ATCC 33277 played a role as a repressor in the oxidative stress response, with genes encoding AhpC, GroES, ClpB, and DnaK included in its regulon [3]. The open reading frame of RprY (PGN_1186 in ATCC 33277 and PG1089 in strain W83) encodes a 28 kDa protein with 61% identity and 74% similarity to a previously identified RprY response regulator from *Bacteroides fragilis* [4]. The amino acid sequence of RprY indicates that it is a member of the OmpR/PhoB superfamily of regulators that act as repressors [5].

In *B. fragilis* and other Bacteroidetes, including the oral species *Prevotella intermedia* and *Tannerella forsythia, rprY* is adjacent to *rprX*, the HK partner. However, an *rprX* homologue is not present in *P. gingivalis*, and there are no proteins with any similarity in this species. Although it is possible that phosphorylation of RprY may be carried out by one of the other HKs present in the *P. gingivalis* genome, we were intrigued by the presence of a protein acetyltransferase (*pat*) gene immediately downstream from *rprY*, and the possibility that protein acetylation may play a role in the regulation of RprY activity. In the present study, we show that *rprY* and *pat* are co-transcribed, and that RprY is acetylated *in vivo*. We also show that RprY is autoacetylated *in vitro* by a reaction in which acetyl CoA is the acetyl donor, and that autoacetylation is enhanced by Pat and reversed by the CobB deacetylase. Furthermore, it appears that phosphorylated, that is, activated RprY is the preferred substrate for acetylation suggesting cross-talk between the protein modification activities. Finally, we show that acetylated RprY had diminished ability to bind to promoter DNA; thus, modification of regulator proteins by acetylation appears to be another mechanism to modulate their function [6], including derepression.

## Materials and methods

### Strains and growth conditions

All bacterial strains used are listed in Table 1. Strains of *Escherichia coli* were derivatives of K12 that were grown in Luria Bertani broth or plates at 37°C, and when appropriate with the addition of antibiotics (ampicillin 100 μg/ml or kanamycin 50 μg/ml). Cultures of *P. gingivalis* strain ATCC 33277 were grown in tryptic soy broth (TSB) as described previously [3] with erythromycin (5 μg/ml) or tetracycline (2 μg/ml) when appropriate. Cultures were grown in an anaerobic chamber (Coy) in an atmosphere of 85% nitrogen, 5% hydrogen, and 10% carbon dioxide.

**Table 1.** Bacterial strains and plasmids used in this study.

Strains	Description	Source
*P. gingivalis*		
ATCC 33277	Type strain	
*pat* mutant	ATCC33277 *Δ1185::ermF-ermAM*	This study
*cobB* mutant	ATCC33277 *Δ0004::ermF-ermAM*	This study
*rprY* mutant	ATCC33277 *ΔrprY::TetQ*	Duran-Pinedo et al. [10]
*E. coli*		
DH5α	*F^−^ φ80dlacZΔM15 Δ(lacZYA-argF)U169 deoR recA1 endA1 hsdR17(rk^−^, mk^+^) phoA supE44 λ^−^ thi-1 gyrA96 relA1*	Invitrogen
BL21(DE3)	*F^−^ ompT hsdS B(rB^−^mB^−^)dcm gal* (DE3)	Novagen
Plasmids		
pET28a	Kan^r^ expression vector with 6His-tag coding sequence	Novagen
pET22b	Amp^r^ expression vector with 6His-tag coding sequence	Novagen
pETRprY	pET28a derivative for expression 6His-RprY	This study
pETPat	pET22b derivative for expression 6His-Pat	This study
pETCobB	pET22b derivative for expression 6His-CobB	This study

**Table 2.** Primers used in this study.

Primer name	Sequence (5’ to 3’)	Used in
Actpet F	TATA*CATATG*CTTCGATTTTCTGACTTAAAGTC	ORF cloning
ActPetR	CTCGAGATGTGGGCAATTATAGACTGCTC	ORF cloning
PGN0004cobB-F	CCCCCATATGAATAAGAAAAGACTCGTCGTCTTGAGT	ORF cloning
PGN0004cobB-R	AAACGGATCCGAGCGATCTATATCACGGAGTTCTTGC	ORF cloning
RprYpet-F	CATATGGAAGAAAAAACAAGAATC	ORF cloning
RprYpet-R	AAGCTTGACCTCTTTGATTGCC	ORF cloning
kar6B	ATCAGGTATGATTACACTGA	RT-PCR
rprYQC6	CTCGGACTGGTGCGCTGCTC	RT-PCR
RprYQc1	CAAGTATATGCTCTACTACC	RT-PCR
RprYQc2	CAGACCTCTTTGATCAACTC	RT-PCR
rprYqc3	GAATCTTTCTCTGCGAGGAC	RT-PCR
rprYqc4	CGTCCGCCACAGCGTCGCAG	RT-PCR
rprYqc5	CTCATTCCGTTCTCAGCTGG	RT-PCR
KK110	GTCAGGGGAGGCTCTGCTTC	RT-PCR
1185RACEout	CCGCACGTAAGAAATTGTCATACGA	5ʹ-RACE
1186Raceout	GATGCCATCCTTACGTGGCATCA	5ʹ-RACE
1186 RACEinn	CGATATTACTATCGTCCTCGCAGAG	5ʹ-RACE
1185 RACE inn	AAGTCAGAAAATCGAAGCATTG	5ʹ-RACE
Race adapter primer	CGCGGATCCGAACACTGCGTTTGCTGGCTTTGATG	5ʹ-RACE
3way- actF1	CGATAGGGTAGGATATTATTGTC	*pat* mutant construction
3way-actR2	GGAAATGGATCGGCATGGTTCTG	*pat* mutant construction
3way-actR1	tgtagataaattattaggtatactactgacagcttcAAGTCAGAAAATCGAAGCATTG	*pat* mutant construction
3way-act F2	accgatgagcaaaaaagcaatagcggaagctatcggTTAATCAGCTTTTGAGAAACAC	*pat* mutant construction
ERM-ACT R1	CAATGCTTCGATTTTCTGACTTGAAGCTGTCAGTAGTATACCTAATAATTTATCT ACA	*pat* mutant construction
ERM-ACTF2	GTGTTTCTCAAAAGCTGATTAACCGATAGCTCCTGCTATTGCTTTTTTGCTCATCGGT	*pat* mutant construction
nqrApF	GACACAGAATTATTATTC	EMSA
nqrApR	ACTCTGCACAGGATGGGA	EMSA
nqrArtF	ACACCGGATATAGCTCCACG	qRT-PCR
nqrArtR	GGACTTCAGGCCCACATAGA	qRT-PCR
rprYrtF	GCGGACGTTCGAACAAGTAT	qRT-PCR
rprYrtR	GTTGTCATCCGCCCATATCG	qRT-PCR
patrtF	CGGGCAGGCTTGTATTGAAA	qRT-PCR
patrtR	CATCAAGTACAGCGGCACTC	qRT-PCR
cobBrtF	AATCCTGCCCTCGTTCTCAA	qRT-PCR
cobBrtR	CTCCGTGCAGATGAATGACG	qRT-PCR
oxyRrtF	CACATCGTCTCTGGCTGTTG	qRT-PCR
oxyRrtR	TAATTCCCTGACCGCTCTCC	qRT-PCR

### Mutant strains and plasmid constructions

Plasmids and primers used in this study are listed in Table 1 and Table 2. DNA primers were synthesized by integrated DNA Technologies. The *rprY* (PGN_1185) deletion strain of ATCC 33277 was constructed as described earlier [3]. Briefly, using three-way SOEing (splicing by overlap extension) [7], PCR amplicons containing sequences 1054 bp upstream and 1047 bp downstream of the PGN_1185 ORF were ligated to an ErmF-ErmAM cassette. The fragment obtained was transferred into ATCC 33277 electrocompetent cells, and transformants were selected for erythromycin resistance. Similarly, a *pat* mutant was constructed that contained the tetracycline resistance gene from pFD288L [8].

### Cloning, expression, and purification of recombinant proteins

*P. gingivalis* genes including *rprY, pat*, and *cobB* were amplified from genomic DNA using gene-specific primers (Table 2). These genes were further cloned into overexpression vector pET22b to produce recombinant plasmids. *E. coli* BL21 (DE3) cells transformed with recombinant plasmids were grown at 37°C in 1L of LB medium containing 100 μg/ml ampicillin. Protein purifications and determinations of concentration were carried out as described previously [3].

### RNA isolation and RT-PCR

*P. gingivalis* strains were grown in TSB broth to A550nm 0.4–0.6 and cells were centrifuged and resuspended in RNA Later (Ambion) and stored at –80°C. RNA was isolated using a DNA/RNA isolation kit (Epicenter), and contaminating DNA was removed by treatment with Turbo DNase (Ambion). The resulting RNA preparation was monitored for DNA contamination by PCR, and the integrity of RNA was analysed visually by gel electrophoresis. Moreover, RNA concentration was measured using a Nanodrop 1000 spectrophotometer (Fisher Scientific). cDNA synthesis was performed with the Revertaid reverse transcription kit (Fermentas) according to the manufacturer’s instructions; briefly, 100 ng of RNA was reverse transcribed for 1 h at 42°C.

### 5ʹ-RACE

The transcription start site of the *rprY* and *pat* genes was determined using the FirstChoice 5ʹ-RLM-RACE kit (Ambion) as described previously [9]. Briefly, 5 μg of RNA was treated with tobacco acid pyrophosphatase (TAP) followed by ligation of the 5ʹ-RACE adapter and cDNA synthesis using PGN_1185 (*pat*) or PGN_1186 (*rprY*) gene-specific primers (Table 2). The cDNA was used for nested PCR using gene-specific and RACE adapter primers to determine the transcription starts (TS) of both genes. The amplification products were ligated to the pJET cloning vector for sequencing.

### Western blotting

*P. gingivalis* ATCC 33277 cultures were grown in TSB to A550nm 0.4–0.6 and processed as described above. Cells were mixed with equal amounts of Laemmli sample buffer (BioRad) and boiled for 10 min. Samples were fractionated on precast 4–20% gradient polyacrylamide gels (Thermo Fisher) and resolved proteins were electrotransferred to nitrocellulose membranes. After transfer, membranes were blocked with 5% non-fat milk dissolved in Tris-buffered saline (TBS) containing 0.1% Tween-20. Primary antibodies, anti-acetylated protein antibody (1:1000, Abcam Ab193), or rabbit anti-RprY antibody [10] were added to membranes followed by overnight incubation at room temperature. After thorough washing, horseradish peroxidase-(HRP) conjugated goat-anti-rabbit secondary antibody was added and the signal was detected using a Western detection kit (Millipore).

### In vitro acetylation and deacetylation assays

For *in vitro* acetylation of RprY (AcRprY), a reaction mix (25 μl) containing N-terminal His6-RprY (100 μM, unless otherwise stated), acetyl-CoA (Sigma Aldrich; 600 μM, unless otherwise stated), and Tris-HCl buffer (10 mM, pH 8.0) was incubated for the indicated times at 37°C. The reaction was stopped by the addition of SDS sample buffer and fractionated on a 12% SDS-polyacrylamide gel. To study the effect of Pat on RprY acetylation, the assays were performed as described above in the presence of Pat at the indicated concentrations. Deacetylation of AcRprY by CobB-His6 (50 μM) was performed in 10 mM Tris-HCl buffer (pH 8.0) in the presence or absence of NAD^+^ (1.5 mM). Specific protein concentrations and reaction times are indicated in Results.

### Dephosphorylation of RprY and Phos-tag gels

To analyse the effect of Ac-CoA on the phosphorylation of RprY *in vitro*, we used Phos-tag acrylamide (Wako Chemicals) to separate phosphorylated and unphosphorylated forms of RprY. RprY was diluted to 20 μM in kinase buffer (20 mM Tris-HCl at pH 8.0, 250 mM NaCl, 10 mM MgCl_2_, 2.5 mM MnCl_2_), and 0.5–1 μM Ac-CoA was added. The reactions were incubated at 37°C for 15 h before quenching by the addition of Laemmli buffer. Dephosphorylation of RprY by Antarctic phosphatase (New England Biolabs) was used as a positive control. The reactions were further fractionated by Phos-tag SDS-PAGE (10% acrylamide, 375 mM Tris-HCl at pH 8.8, 0.1% SDS, 50 μM Phos-tag, 150 μM MnCl_2_). Gels were run at 100 V, 120 min on ice. Prior to transfer to PVDF membranes, gels were incubated with transfer buffer containing 20% methanol and 1 mM EDTA for 20 min. After transfer, proteins were visualized using RprY specific primary and goat anti-rabbit HRP-conjugated secondary antibodies (Pierce).

### EMSA

The promoter of PGN_0118 (*nqrA*) was amplified by PCR from strain ATCC 33277 chromosomal DNA and labelled with the DIG Gel Shift Kit (Roche) according to the manufacturer’s instructions. The EMSA reaction mix (20 μl) contained 0.80 pmol/ml DIG-labelled DNA and various amounts of RprY diluted in a buffer containing 50 mM Tris-HCl (pH 8.0), 750 mM KCl, 2.5 mM EDTA, 0.5% Triton-X, 2.5% glycerol, and 1 mM DTT. After incubation for 30 min at room temperature, the reactions were mixed with Hi-Density TBE Sample Buffer (Invitrogen), loaded onto native 6% polyacrylamide precast gels. Gels were run at 80 V, 120 min on ice; then, DNA substrates and DNA-protein complexes were electrotransferred to positively charged nylon membranes (GE Health Care) and incubated with anti-digoxigenin antibody (Roche). Detection with CSPD (Roche) was carried out according to the manufacturer’s instructions.

### In vitro chromatin immune-precipitation (ChIP)

The binding of RprY to sonicated genomic DNA fragments (300 bp–500 bp) of strain ATCC 33277 was measured by *in vitro* ChIP assays. Purified His-RprY (20 μM) was pre-incubated with Ac-CoA (400 μM) or AcP (400 μM) at 37°C for 15 h. The pre-treated RprY was further incubated with Ni^+^-NTA resin (Thermo Fisher) in 500 μl binding buffer (50 mM NaH_2_PO_4_, 50 mM NaCl, 20 mM imidazole, pH 8.0) at 4°C for 1.5 h. Ni**^+^**-NTA resin without RprY was used as a negative control. Sonicated ATCC 33277 genomic DNA (10 μg in 50 μl buffer) was mixed with Ni**^+^**-NTA in a 500 μl reaction buffer (20 mM Tris-HCl, 10 mM MgCl_2_, 100 mM KCl, 20 mM imidazole, pH 8.0) and incubated at room temperature, 30 min. DNA bound to the beads was eluted with 100 μl of 1.25 M NaCl. The concentration of eluted DNA was measured by a picoGreen kit (ThermoFisher) according to the manufacturer’s instructions.

### RNA isolation for qRT-PCR assays

ATCC 33277 *rprY* and *pat* mutant strains were grown in TSB to exponential growth phase (A550nm 0.4–0.6). Cultures were split into two equal parts, centrifuged, and resuspended in pre-reduced TSB with or without NaCl. Cells were further incubated for 2 h anaerobically before harvest. RNA was immediately isolated from cell pellets using the MasterPure RNA purification kit (Epicentre) according to the manufacturer’s instruction. DNA contamination was eliminated by treatment with Turbo DNase (Ambion). Isolated RNA was monitored for DNA contamination by PCR. RNA integrity was visually assessed after gel electrophoresis. The concentration and purity of RNA were determined using a Nanodrop 8000 Spectrophotometer (ThermoFisher).

For qRT-PCR, RNA (100 ng) was reverse transcribed with a RevertAid First strand cDNA synthesis kit (Fermentas) according to the manufacturer’s instructions. Gene-specific primers were designed using the Primer3 online tools (http://simgene.com/Primer3). A three- step protocol was used for PCR amplification: 95°C for 5 min, and 45 cycles at 95°C for 15 s, 55°C for 15 s, and 72°C for 15 s. Amplification specificity was assessed by conducting a melting curve analysis. Gene expression levels were normalized to those of 16S rRNA transcripts. Relative fold-changes in expression were calculated using the 2^−ΔΔCt^ method [11].

## Results

### PGN_1185 and PGN_1186 are encoded in an operon

**Figure 1.**
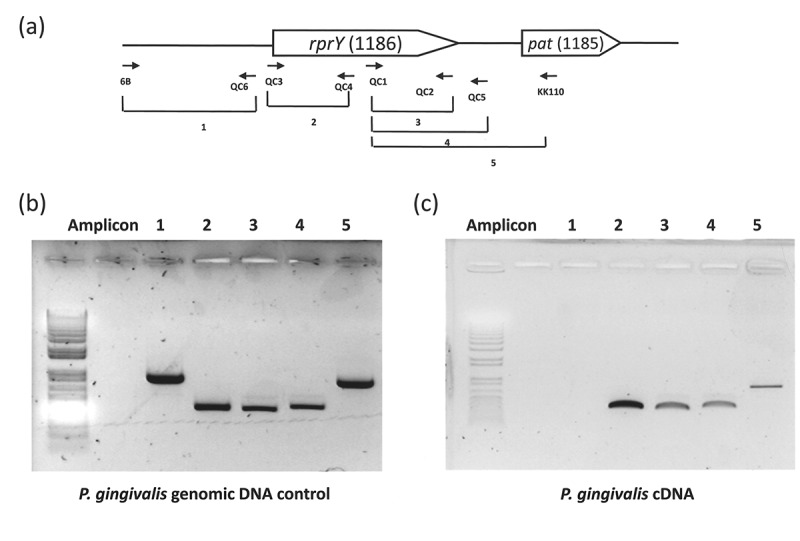
*rprY* and *pat* are co-transcribed as an operon in *P. gingivalis* ATCC 33277. (a) Schematic of the *rprY-pat* locus in strain ATCC 33277. Locations of RT-PCR primers are indicated by small arrows and corresponding amplicons are numbered. (B and C) RT-PCR assays for expression of *rprY-pat* genes. (b) *G*enomic DNA was used as the positive control template for PCR. Amplicon numbers correspond to those indicated in panel A. (c) cDNA was used as the template for PCR.

The genetic locus and context of response regulator *rprY*, PGN_1186, in the genome of *P. gingivalis* strain ATCC 33277 is shown in Figure 1(a). In two-component systems, HK (sensor) and cognate RR genes are most often encoded in an operon; however, there are also examples of the genes located at different loci in the genome. RprY is considered an orphan regulator because the cognate HK is not encoded upstream or downstream of the RR; thus, the gene(s) responsible for the activation of *rprY* is not known. In addition, shown in Figure 1(a), is the gene downstream from *rprY*, PGN_1185, which is annotated as an acetyltransferase of the GNAT family and called *pat* (protein acetyltransferase) for simplicity in this study. BLAST searches indicate the same genetic locus is present in all the sequenced genomes of *P. gingivalis* strains, except that they lie on the minus DNA strand in strains ATCC 33277 and W83 and the plus strand in strains TDC60 and W50.

To determine whether *rprY* and *pat* are transcribed as a monocistronic message, we performed RT-PCR with RNA from strain ATCC 33277 grown under standard broth culture conditions. Different sets of primers were used to determine the co-transcription of PGN_1186 and PGN_1185 (Table 1). Set 1 amplifies the upstream region of the *rprY* ORF (Figure 1(a)), and this was used as the negative control for transcription. This set was able to generate an amplicon from genomic DNA (Figure 1(b)) but not cDNA (Figure 1(c)) indicating a clean preparation of RNA. We were able to detect amplification products of the predicted sizes from the other primer sets. We observed that in spite of the distance between the two adjacent ORFs (116bp) they were encoded as a single RNA transcript.

### Genes for rprY (PGN_1186) and pat (PGN_1185) have the same transcription start (TS)

**Figure 2.**
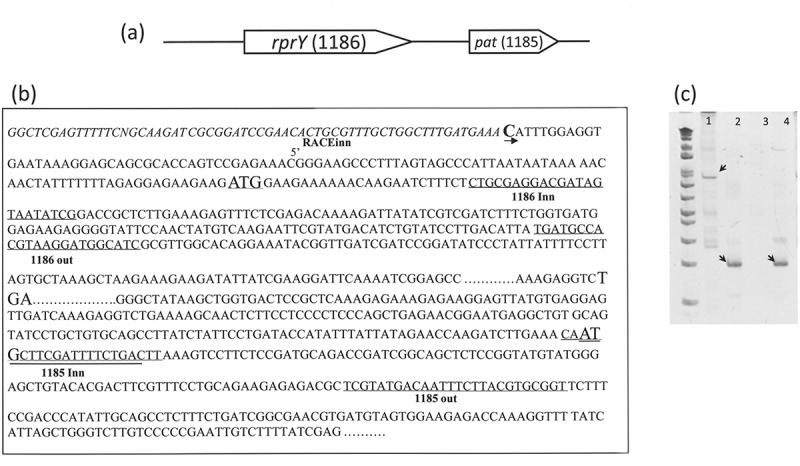
*RprY* and *pat* have the same transcription start. (a) Schematic of the *rprY-pat* locus in strain ATCC 33277. (b) The positions of primers used for 5ʹ-RACE assays are indicated in the sequence of the promoter regions. (c) RACE amplicons (indicated by arrows) were sized relative to the DNA ladder (first lane).

The TS of *rprY* and *pat* (schematic in Figure 2(a)) were determined by 5ʹ-RLM-RACE. Following ligation of the 5ʹ-RACE adapter to the 5ʹ ends of TAP-treated RNA, it was reverse transcribed to cDNA in two different syntheses. First, the specific primer 1185RACEout was used to generate 5ʹ-adapter-linked cDNA of *pat* (Figure 2(b)). To determine the TS of *pat*, this cDNA was used as the template for PCR amplification using the 5ʹ-RACE inner and 1185 inn primer set. The reaction yielded a 1016 bp product (Figure 2(c), lane 1). As an additional confirmation an amplification using the 5ʹ-RACE inner and 1186 inn primer set yielded a 190 bp product (Figure 2(c), lane 2). Sequencing of the amplification products showed that PGN_1185 (*pat*) and PGN_1186 (*rprY*) have the same TS 103 bp upstream of the *rprY* translation start site. For the second cDNA synthesis, the PGN_1186-specific primer (1186 race out) was used to generate the 5ʹ-adapter-linked cDNA for *rprY* (Figure 2(b)). Correspondingly, this cDNA was used as the template for PCR amplification using the 5ʹ-RACE inner and 1186 inn primer set. This reaction also yielded a 190 bp product (Figure 2(c), lane 4) which was sequenced and revealed that the TS of PGN_1186 (*rprY*) was 103 bp upstream of the PGN_1186 translation start site. These data established that both *pat* and *rprY* have the same TS, and confirmed that the genes are encoded in an operon.

### RprY is acetylated in vivo and in vitro

**Figure 3.**
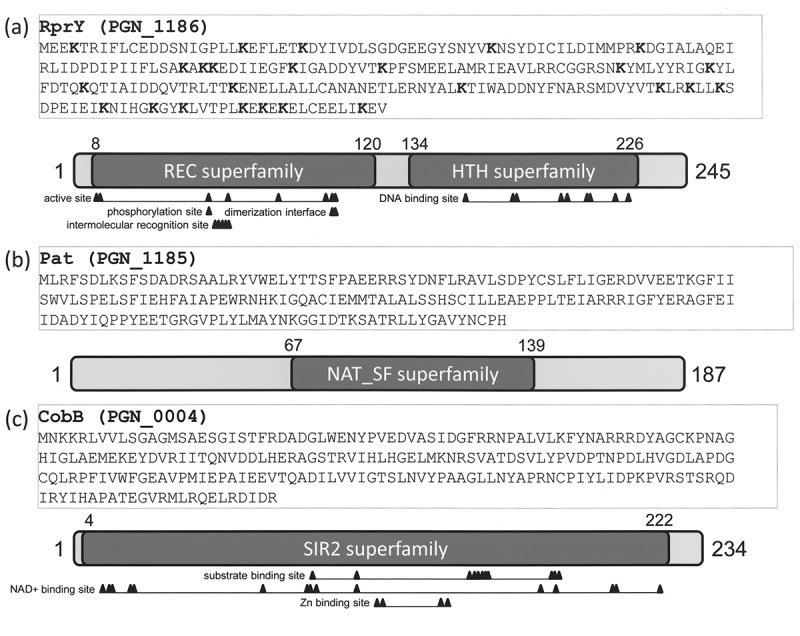
Amino acid sequences and conserved domains of RprY, Pat, and CobB. Conserved domains were predicted by NCBI CDD database.

The RprY sequence (Figure 3(a)) shows the lysine residues (in bold) that are potential sites for Nɛ-acetylation by Pat. The putative phosphorylation sight is D55 [12]. Lysine residues are fairly evenly distributed within the protein but are more clustered at the C-terminus, similar to the sequence in CheY from *E. coli* [13]. The RprY C-terminus contains the DNA binding domain.

The protein sequence of Pat is depicted in Figure 3(b) with the conserved domain of the Gcn5-related acetyltransferase (GNAT) family [12] depicted below. These enzymes catalyse the transfer of an acetyl group from acetyl-CoA to the Nε-amino group of a lysine residue in a protein [14]. This form of acetylation can be reversible and in some cases irreversible [15], and is implicated as a regulatory mechanism that can potentially modulate protein–protein, DNA–protein, and other interactions. How RprY is activated by phosphorylation is still unclear, but the finding that the regulator is co-transcribed with a GNAT acetyltransferase prompted us to investigate whether RprY is acetylated *in vivo* and whether Pat activity is responsible.

**Figure 4.**
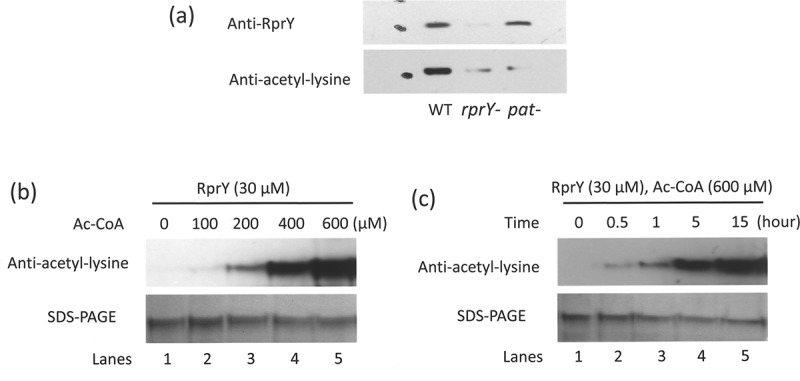
RprY is acetylated *in vivo* and *in vitro*. (a) *In vivo* production of acetylated RprY in parent strain ATCC 33277 (WT) and *rprY* and *pat* mutant strains was detected in Western blots probed with either anti-RprY or anti-acetyl-lysine antibodies. (b) RprY (30 μM) was incubated for 15 h at 37°C with increasing concentrations of Ac-CoA. Acetylation of RprY was detected in Western blots probed with anti-acetyl-lysine antibody. Proteins fractionated by SDS-PAGE and stained with Coomassie indicated equal loading. (c) RprY (30 μM) was incubated with Ac-CoA (600 μM) at 37°C for the times indicated. Acetylated RprY was detected by Western blot as above and RprY protein by SDS-PAGE as above.

Cell extracts prepared from parent, and *rprY* and *pat* deletion mutants were fractionated by SDS-PAGE and transferred to nitrocellulose. The Western blots were probed with anti-RprY and anti-acetyl-lysine antibodies. The Western probed with anti-RprY antibody showed that the protein was present in both the parent and *pat* mutant strains but absent in the *rprY* mutant (Figure 4(a)). The Western probed with anti-acetyl-lysine antibody showed an acetylated band in the parent extract. However, traces of acetylated protein were also observed in the mutant extracts. Although it is possible that this was due to sample overflow from the neighbouring well, we also acknowledge that the Western identified another protein with similar molecular weight to RprY that was also acetylated, but not recognized by anti-RprY antibody. Although further genetic analyses are required to resolve this question, conditions for *in vitro* and *in vivo* acetylation and regulatory properties of RprY are the focus of the present study.

To determine whether RprY could be chemically acetylated *in vitro*, the purified protein was incubated with acetyl-CoA (Ac-CoA) as the acetyl donor. Acetylation of RprY was detected by probing Western blots with anti-acetyl-lysine antibody. As shown in Figure 4(b,c), the addition of acetyl groups to RprY was dependent on both the concentration of Ac-CoA and the length of incubation.

### Effects of Pat and CobB on in vitro acetylation of RprY

**Figure 5.**
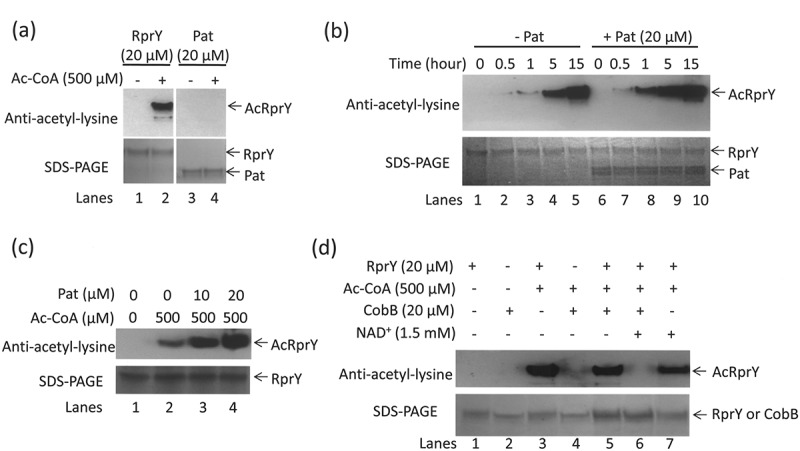
Effects of Pat and CobB on *in vitro* acetylation of RprY. (a) RprY (20 μM) or Pat (20 μM) were incubated for 15 h at 37°C with 500 μM AcCoA. Acetylated proteins (indicated by arrows) were detected in Western blots probed with anti-acetyl-lysine antibody. Proteins were also Coomassie-stained after SDS-PAGE. (b) Effect of Pat on the acetylation of RprY. Acetylation reactions were carried out in the absence or presence of Pat for the indicated times and concentrations of Ac-CoA. Western blots were probed with anti-acetyl-lysine antibody, and proteins were visualized by Coomassie stain. (c) Reactions were also carried out with increasing amounts of Pat (0, 10, and 20 μM) in the presence of RprY (20 μM) and 500 μM Ac-CoA for 5 h. (d) Effect of CobB on the acetylation of RprY. Acetylation reactions contained 20 mM RprY, 20 mM CobB, 500 mM Ac-CoA, and 1.5 mM NAD^+^ (CobB cofactor). Acetylated RprY was detected in Western blots probed with anti-acetyl-lysine antibodies. Proteins in reactions were stained with Coomassie.

Because *pat* and *rprY* are co-transcribed, we tested the effect of recombinant Pat on the acetylation of RprY *in vitro*. First, we established that RprY was autoacetylated in the presence of 500 μM Ac-CoA, while Pat was not (Figure 5(a)). As shown in Figure 5(b,c), the acetylation level of RprY increased when Pat (5–20 μM) was added to the reaction mix, indicating that Pat enhanced the autoacetylation of RprY. Based on these observations, we hypothesized that Pat functioned as the modifier of RprY protein *in vitro*. These results also suggest that autoacetylated RprY does not transfer acetyl groups to other proteins, indicating that RprY itself does not possess an acetyltranferase activity.

CobB, PGN*_*0004 in strain ATCC 33277, is a Sir2 family nicotinamide adenine dinucleotide (NAD^+^)-dependent deacetylase (Figure 3). To test whether RprY was a substrate for recombinant CobB deacetylase activity, the acetylation levels of RprY in the presence of CobB were analysed. Acetylation of RprY was not affected in the absence of CobB or NAD^+^ (Figure 5(d), lanes 5, 7), but was significantly reduced in the presence of CobB and NAD^+^ (Figure 5(d), lanes 6). We also found that CobB was not autoacetylated in the presence of Ac-CoA (Figure 5(d), lane 4). These results clearly demonstrate that CobB deacetylates RprY *in vitro*.

### Cross-talk between acetylation and phosphorylation of RprY

**Figure 6.**
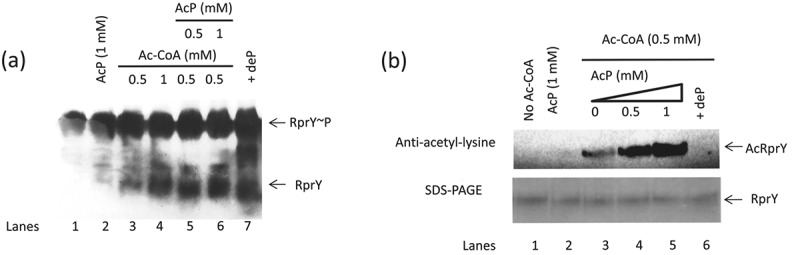
Cross-talk between acetylation and phosphorylation of RprY. (a) RprY was phosphorylated by treatment with AcP (1 mM) followed by incubation with Ac-CoA (0.5 and 1 mM) in the absence or presence of AcP (0.5 and 1 mM). Reaction mixes were fractionated on Phos-Tag gels to separate phosphorylated and dephosphorylated forms of RprY as indicated by arrows. RprY~ P was treated with Antarctic phosphatase as a positive control for dephosphorylation (dep). (b) Western blots showing effects of AcP and phosphatase on acetylation of RprY. Acetylation reactions were carried out with the indicated concentrations of Ac-CoA and AcP for 5 h at 37°C.

Next, we determined the effect of autoacetylation on the phosphorylation (activation) of RprY. Phos-tag gels separate phosphorylated from unphosphorylated forms of proteins [16], a property we validated by using Antarctic phosphatase-treated recombinant RprY as a positive control (Figure 6(a), lane 7); this treatment also confirmed that RprY purified from *E. coli* was already phosphorylated (Figure 6(a), lanes 1 and 7). Phosphorylated RprY was incubated with Ac-CoA to examine whether acetylation affected phosphorylation, and as shown in Figure 6(a) (lanes 2–4), the increased levels of non-phosphorylated RprY in Phos-tag gels in the presence of increasing amounts of Ac-CoA indicated that acetylation of RprY resulted in dephosphorylation, and possibly inactivation, of the regulator. The additional presence of acetyl phosphate (AcP) appeared to limit dephosphorylation but not reverse the reaction (Figure 6(a), lanes 5 and 6). Conversely, results from western blots of assays to examine the effects of AcP on acetylation of RprY suggested that phosphorylation of the protein enhanced acetylation (Figure 6(b)). Clearly, the potential interplay between acetylation and phosphorylation of RprY warrants future investigation.

### Acetylation modulates the interaction of RprY with a target promoter and reduces RprY phosphorylation

**Figure 7.**
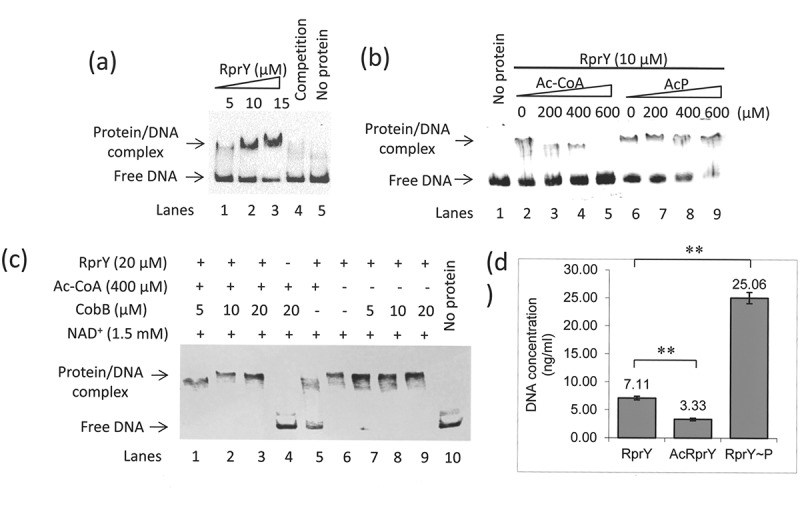
Effects of acetylation and phosphorylation on the DNA binding activity of RprY. (a) EMSA showing binding of RprY to the *nqrA* promoter. The reactions contained DIG-labelled DNA (0.5 pmol) from the *nqrA* promoter region and increasing amount of RprY (5 μM, 10 μM, and 15 μM). Excess unlabelled *nqrA* promoter was used as competitor to test the specificity of DNA binding. The protein/DNA complexes are indicated by arrows on the left of the panels. (b) Effects of Ac-CoA and AcP on the DNA binding activity of RprY were measured by EMSA. The concentration of RprY in lanes 2–9 was 10 μM. The increasing concentrations of Ac-CoA or AcP are indicated at the top of the panels. (c) Effects of CobB (deacetylation) on the DNA binding activity of RprY were measured by EMSA. These reactions were carried out using increasing amounts of CobB in the absence or presence of Ac-CoA. (d) *In vitro* ChIP assays to determine the effect of Ac-CoA and AcP on the interaction of RprY with sonicated ATCC 33277 genomic DNA (average size 300–500 bp). RprY (20 μM) in the *in vitro* ChIP assays was pre-incubated with Ac-CoA (500 μM) or AcP (500 μM) for 15 h at 37°C. The experiment was carried out as described in Materials and methods. The concentration of DNA recovered from the immunoprecipitates was measured using PicoGreen reagents. Values represent the means ± standard deviations from three independent experiments. Asterisks indicate statistically significant differences compared to the untreated RprY control (**P < 0.01).

We investigated the effects of acetylation and phosphorylation on the ability of RprY to bind to the promoter of *nqrA* (PGN_0115: Na**^+^**-translocating NADH-quinone reductase subunit A) which we previously showed was a target of regulator RprY [10]. First, in electromobility shift assays (EMSA), we showed that RprY bound to the DIG-labelled *nqrA* promoter DNA in a concentration dependent manner and that addition of excess unlabelled promoter DNA competed for binding indicating specificity (Figure 7(a)). Next, we showed that the addition of increasing concentrations of Ac-CoA reduced the formation of RprY-DNA complexes (Figure 7(b), lanes 2–5), with no detectable shift in mobility at the highest Ac-CoA concentration (600 mM). On the other hand, the presence of increasing concentrations of AcP resulted in increased incorporation of the labelled promoter into RprY-DNA complexes (Figure 7(b), lanes 6–9).

To further address the question of reduced DNA binding by acetylated RprY, we carried out EMSA in which RprY was acetylated by Ac-CoA in the presence or absence of recombinant CobB deacetylase. EMSA with CobB and cofactor NAD^+^ showed shifted RprY-DNA complexes (Figure 7(c), lanes 1–3). A control reaction with CobB in the absence of RprY did not show shifted complexes (Figure 7(c), lane 4) and acetylated RprY in the absence of CobB showed reduced complex formation (Figure 7(c), lane 5). We also showed that the presence of CobB deacetylase did not prevent the formation of unacetylated RprY-DNA complexes (Figure 7(c), lanes 7–9). Finally, *in vitro* ChIP assays were carried to quantify binding of non-acetylated and acetylated RprY to sonicated genomic DNA fragments of *P. gingivalis*. The binding of DNA to acetylated RprY was reduced approximately twofold compared to the unacetylated RprY control, while phosphorylation of RprY increased DNA binding at least threefold compared to the control. In conclusion, these results indicate that the DNA binding ability of RprY was regulated by both acetylation and phosphorylation, and specifically that acetylation of RprY interfered with its ability to bind to target promoter DNA that consequently may alter gene expression.

### RprY acetylation and gene expression under Na^+^-depleted conditions

**Figure 8.**
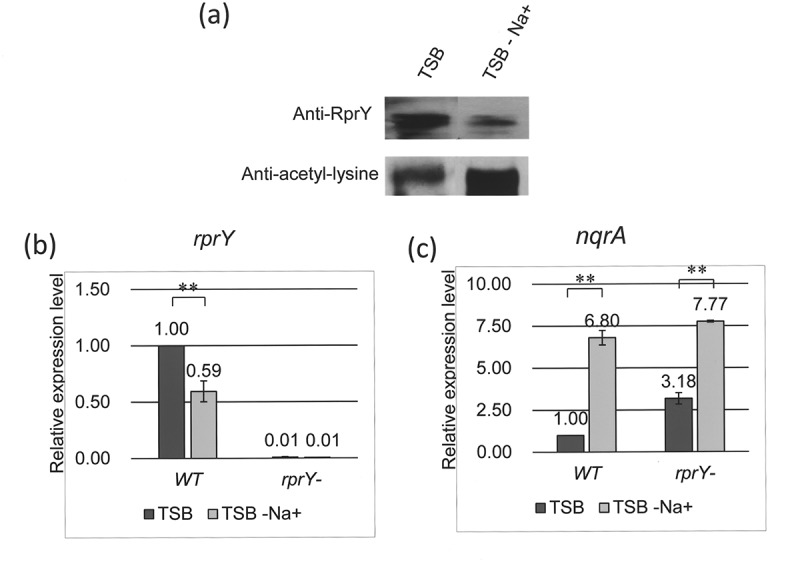
RprY acetylation and gene expression under Na^+^-depleted conditions. (a) Western blot showing expression and acetylation of RprY in strain ATCC 33277 incubated in TSB with and without Na^+^. (b and c) Expression of *rprY* (b) and *nqrA* (c) genes in ATCC 33277 parent and *rprY – mutant* strains incubated in TSB with and without Na^+^. Expression was measured by qRT-PCR, and values were normalized to those of 16S rRNA gene transcripts and fold-changes in expression were calculated. Values represent the means ± standard deviations from three independent experiments. Asterisks indicate statistically significant differences compared to expression levels in TSB medium (*, *P *< 0.05; **, *P *< 0.01).

Previously, we demonstrated that Na**^+^**-depleted culture conditions induced the expression of an *rprY* promoter-LacZ fusion protein in *E. coli*, and led to a hyper-oxidative stress response in a *P. gingivalis rprY* mutant that severely limited growth [3]. In light of these observations, we investigated the effects of Na**^+^** depletion on production and acetylation of RprY. Cell extracts were prepared from strain ATCC 33277 cells incubated in complete TSB and resolved by SDS-PAGE. RprY was detected in Western blots probed with anti-RprY and with anti-acetyllysine antibodies (Figure 8(a)) again indicating that RprY was acetylated *in vivo*. In extracts from the same strain incubated in Na**^+^**-depleted TSB, the level of RprY protein was reduced by at least 60% but was highly acetylated compared to that from cells in normal TSB.

To determine whether the function of RprY as a repressor was impaired in ATCC 33277 parent cells harvested from Na**^+^**-depleted TSB, we measured the relative expression of two promoter targets of RprY, that is, *nqrA* and *rprY* itself. By qRT-PCR, expression of *rprY* was reduced approximately 40% in cells from Na**^+^**-depleted TSB (Figure 8(b)), consistent with the Western data. No expression was detected in the *rprY* mutant (negative control). Our working model is that RprY is a repressor and when the acetylated protein cannot bind to promoter targets repressor function is lost. Consistent with this model are the effects of Na**^+^** depletion on *nqrA* expression which is almost seven times that in cells from normal TSB (Figure 8(c)). The data from the *rprY* mutant are similar in that *nqrA* expression levels are higher than in the parent strain, even in cells from normal TSB, supporting the model that RprY is a repressor. In addition, *nqrA* expression increases over twofold in the *rprY* mutant from Na**^+^**-depleted TSB, suggesting that NqrA function is involved in the response to this stress.

## Discussion

In all *P. gingivalis* strains sequenced so far RprY is an ‘orphan’ RR because a cognate HK has not yet been identified. In place of a neighbouring HK, we identified the contiguous gene as a protein acetyltransferase (*pat*) of the GNAT family based on protein homology. The findings that *pat* (PGN_1185) was co-transcribed with *rprY* (PGN_1186) and had the same transcriptional start (Figure 1) prompted the present study to determine whether acetylation, a post-translational modification, altered the regulatory functions of RprY in strain ATCC 33277. Acetylation was originally identified as a modification of histone proteins in eukaryotes, but recently similarly acetylated proteins were identified in prokaryotes reviewed by Soppa [17] and Jones and O’Connor [18]. Nε-Lysine acetylation of proteins is effected by acetyl CoA synthase and a Gcn-5-like acetyltransferase (GNAT) that uses acetyl CoA as the acetyl donor with the concomitant release of CoA [19]. The modification is reversed by a deacetylase, and the bacterial CobB sirtuin was identified as responsible for this function [20]. Based on protein homology, we identified PGN_0004, annotated as an NAD-dependent deacetylase, as CobB with 58% exact and 73% positive identity to the CobB protein from *Bacteroides thetaiotaomicron* (accession number NP_811887).

We detected acetylated RprY in *Pg* ATCC 33277 parent cell extracts after Western blotting and probing with anti-acetyl-lysine antibodies, and while the *pat* mutant produced RprY as detected with anti-RprY antibody, the protein was not detected by the anti-acetyl-lysine antibody (Figure 4(a)). These data indicated that RprY was acetylated *in vivo* with the involvement of Pat activity. Several studies showed that proteins can be chemically acetylated with either acetyl CoA or acetyl phosphate *in vitro* [21–23]. To separate protein acetylation from phosphorylation functions, we chose to examine acetyl CoA as acetyl donor. Western blotting using anti-acetyl-lysine antibodies and protein visualization by Coomassie staining showed that acetylation of RprY was dependent on the concentration of acetyl-CoA and the length of incubation (Figure 4(b,c)). These properties, including retention of the modification after SDS-PAGE, suggest that RprY is autoacetylated covalently [21] and specifically, since Pat itself is not acetylated under the same conditions (Figure 5(a)), but does appear to enhance the acetylation of RprY in the presence of acetyl CoA (Figure 5(b,c)). The CobB deacetylase recognized autoacetylated RprY as a substrate, and in the presence of cofactor NAD^+^ removed the modification (Figure 5(d)). A recent study showed that CobB did not deacetylate all acetyl-lysines in proteins whether derived enzymatically or non-enzymatically (autoacetylated) with Ac-CoA or AcP [15]. However, further analyses indicated that the most common CobB sensitive targets were metabolic enzymes and binding proteins, including those involved in DNA binding, transcription, and phosphorylated proteins [15,22]. Indeed, our data show that, in the presence of Ac-CoA and NAD^+^, CobB efficiently deacetylated RprY (Figure 5(d), lane 6).

Protein acetylation in prokaryotes is a relatively new field of study and this post-translational modification affects many classes of proteins including the enzymes of central metabolism, ribosomal proteins, as well as certain transcription regulators. Acetylated proteins are more abundant in bacteria in the stationary phase of growth, and after resuspension of such cells in fresh medium the number of acetylated proteins is reduced [24]. A recent report on the acetylome of *P. gingivalis* affirms that many proteins of varied function, predominantly in metabolism, are acetylated, and suggests that this modification may play a role in pathogenesis [25]. Data from a genetic study in *P. gingivalis* suggested that the *vimA* gene encodes a putative acetyltransferase that may regulate maturation of gingipain cysteine proteinases and possibly other factors associated with virulence [26].

Many functions are assigned to acetylation, for example, protein stabilization, and in the case of transcription regulators it was hypothesized that in eukaryotes the modification alters the charge of the lysine amino group decreasing the affinity of the regulator for target promoter DNA. This notion was validated in the cases of the CheY and RcsB response regulators of *E. coli* [6] and [13], respectively). In the case of CheY, it was revealed by co-crystallization that certain lysine residues were directly involved in binding to DNA targets [27,28].

It has been suggested that cross-talk between protein modifications exists in prokaryotes as in eukaryotes [29]. Therefore, we began a series of experiments to investigate potential cross-talk between acetylated and phosphorylated versions of RprY. The results revealed that the presence of increasing concentrations of Ac-CoA lead to dephosphorylation of RprY (Figure 6(a), lanes 2–4) and addition of AcP appeared to arrest but not reverse dephosphorylation (Figure 6(a), lanes 5 and 6). Interestingly, an additional experiment suggested that phosphorylation of RprY may enhance acetylation (Figure 6(b), lanes 3–5). Although these experiments clearly indicate that cross-talk exists between the modifications, we cannot rule out the possibility that the acetyl phosphate used as the phospho-donor was also able to acetylate RprY. In fact, it was reported that in *E. coli* there is a second non-enzymatic mechanism with acetyl phosphate generated from glycolysis [22,30]. Although beyond the scope of the present study, future experiments will be carried out to determine whether acetyl phosphate can also acetylate RprY.

Our main interest was whether acetylation altered the function of RprY as a regulator, that is, its ability to bind to the promoter DNA of a known target gene, *nqrA*. EMSA were used to detect binding in the presence or absence of AcP or Ac-CoA, and in the presence of the CobB deacetylase. These assays indicated that acetylated RprY showed reduced formation of RprY-*nqrA* DNA complexes (Figure 7(b), lanes 2–5) while DNA binding increased with increasing concentrations of AcP (Figure 7(b), lanes 6–9). In addition, Ac-CoA auto-acetylated RprY is deacetylated by the presence of CobB and NAD^+^ and so is able to bind to the *nqrA* promoter (Figure 7(c), lanes 1–3). When CobB is omitted from the reaction, only a fraction of the DNA is shifted showing that acetylation affects binding (Figure 7(c), lane 5), and CobB itself does not affect the binding of unacetylated RprY to promoter DNA (Figure 7(c), lanes 7–9). Data from *in vitro* ChIP assays support the proposal that acetylation interferes with DNA binding because phosphorylated RprY bound more than three times as much genomic DNA as the acetylated regulator (Figure 7(d)). Finally, we showed an *in vivo* consequence of the reduced binding acetylated of RprY to promoter targets. We know from previous work that growth of strain ATCC 33277 in Na**^+^**-depleted medium affects expression of genes in the RprY regulon [3]. Therefore, we examined whether this condition affected expression and acetylation of RprY itself, and consequently the expression of the target gene *nqrA*. Under Na**^+^** depletion conditions, RprY production was reduced and the protein was more highly acetylated, a result that was confirmed by QRT-PCR (Figure 8(a,b)). The resulting increase in *nqrA* gene expression confirms the repressor function of RprY (Figure 8(c)).

Most of the data reported in this study were obtained with recombinant RprY that was autoacetylated *in vitro* by treatment with Ac-CoA, so how relevant are these finding to the *in vivo* state and function of the regulator? We know that RprY is acetylated *in vivo*, and experiments to isolate larger quantities of the native protein for structural studies are planned. The *in vivo* acetylation reaction is mediated by Pat activity donating the acetyl group from acetyl CoA that was generated from pyruvate by pyruvate–flavodoxin oxidoreductase (PGN_1418) activity. In addition, acetyl-CoA can also be generated from acetate via the reversible activities of acetate kinase (PGN_1081) and phosphotransacetylase (PGN_1082). Both the latter were identified as essential genes in strain ATCC 33277 [31] and interestingly are located four genes upstream from *pat* (PGN_1185) and *rprY* (PGN_1186), raising the possibility that they are or once were part of an acetylation unit.

In ATCC 333277, PGN_1185 is annotated as a conserved hypothetical protein, while in TDC6O the same protein (PGTDC60_1120) is annotated as acetyltransferase. By protein BLAST (Basic Local Alignment Search Tool, https://blast.ncbi.nlm.nih.gov/) PGN_1185 has 98–100% amino acid identity to a GNAT acetyltransferase in 13 *P. gingivalis* strains with genome sequences deposited in GenBank [32]. According to HOMD (Human Oral Microbiome Database, http://www.homd.org/), there are 10 acetyltransferases in the ATCC 33277 genome [32]. Of these, six were annotated as follows: a probable serine acetyltransferase (PGN_0230); a metal binding acetyltransferase (PGN_0913); an alginate O-acetyltransferase (PGN_0943); a lipid phospholipid acetyltransferase (PGN_1142); a probable 1-acyl-sn-glycerol 3-phosphate acetyltransferase (PGN_1384); and a lysophospholipid acetyltransferase (PGN_2086). The remaining three (PGN_0932, PGN_1389, PGN_1729) were annotated as GNAT acetyltransferases, and the present study confirms that PGN_1185 is a fourth. A possible fifth is the VimA multifunctional protein (PGN_1056 in ATCC 33277) annotated as virulence modulating gene A [32] and subsequently as an acetyltransferase [26]. The conserved domain of VimA places it in the NAT_SF super family which includes GNAT as well as other enzymes. Previously, it was demonstrated that in *P. gingivalis* strain W50 over 100 proteins of differing functional classes were acetylated *in vivo* [25]. In these experiments to determine the total acetylome of *P. gingivalis*, acetylated RprY was not detected in cell extracts of W50 cells grown under standard laboratory conditions [25], nor in ATCC 33277 (Li et al, unpublished; Li and Duncan, unpublished). One possibility is that these conditions were not optimal for expression of *rprY* and/or the acetylation of a low abundance protein was undetectable compared to that of a large number of highly expressed metabolic proteins. Are any of the five GNAT acetyltransferases located near genes involved in gene regulation or virulence? Most interesting is the GNAT acetyltransferase PGN_1729 in strain ATCC 33277 which is located 5ʹ to the coding sequence of PGN_1728 (*kgp*) encoding lys-gingipain, and also downstream from hemagglutinin A (PGN-1733) which are both implicated in virulence activities [33–35]. Both lys-gingipain and hemagglutinin (PG1844 and PG1837, respectively) are acetylated in strain W50 [25].

The question of how is RprY activated in *P. gingivalis* is still unclear, although it is possible that it is constitutively activated and switched off by acetylation. RprX, the cognate HK, was first discovered in *B. fragilis* [4] and is present in other oral Bacteriodetes genera such as *Prevotella* and *Tannerella*. RprX is also present in several *Porphyromonas* spp., that is, *P. endodentalis* ATCC 35406, *Porphyromonas* sp. oral taxon F0450, *P. asaccharolytica* DSM 20707, and *P. uenonsis* [36]. However, it is not present in the sequenced *P. gingivalis* strains TD60, ATCC 33277, W83, and W50, and instead *rprY* is contiguous to *pat* with *ackA* and *pta* genes upstream. The orientation of this cluster varies between the strains and notably in ATCC 33277 is bounded by *ISPg*1 elements, suggesting IS-mediated transposition into or translocation within the genome. We previously reported that components of the RprY regulon were induced by Na**^+^** depletion which generated a hyper-oxidative stress response in strain ATCC 33277 [3]; however, there was no obvious direct physiological connection between the inducing condition and the response. Interestingly, examination of the *rprx/rprX* locus in *P. asaccharolytica* and *P. uenonsis* revealed that the complete two-component system is adjacent to an Na**^+^**/H**^+^** antiporter (NhaA) and a Na**^+^** H**^+^** ion exchanger, suggesting a previous association with Na^+^ regulation that may have been lost during evolution of *P. gingivalis* strains.
